# MicroRNAs as Potential Serum Biomarkers for Early Detection of Ectopic Pregnancy

**DOI:** 10.7759/cureus.2344

**Published:** 2018-03-19

**Authors:** Emmanuel N Kontomanolis, Sofia Kalagasidou, Zacharias Fasoulakis

**Affiliations:** 1 Obstetrics and Gynecology, Democritus University of Thrace, University Hospital of Alexandroupolis; 2 Department of Obstetrics and Gynecology, Bodosakio General Hospital of Ptolemaida

**Keywords:** ectopic pregnancy, micrornas, early detectio, diagnosis

## Abstract

Diagnosis of ectopic pregnancy relies on both ultrasound findings and human chorionic gonadotropin (hCG) measurements but due to the need for serial tests, tubal rupture and death represent major maternal and fetal risks. Early detection of ectopic pregnancy is essential and thus a noninvasive diagnostic tool seems crucial for the prevention of adverse effects since studies suggest there is a specific relationship between ectopic pregnancy and increasing microRNA factors. Human fluids in women with ectopic pregnancy reveal a particular change in comparison to healthy women. In addition to certain placental microRNAs circulating through plasma that present a specific concentration and serum profile, microRNAs seem to be possible biomarkers for the detection of pregnancy complications linked to placental pathologies. The aim of this study is to review current literature considering the expression levels of several circulating microRNAs that have shown to be novel potential biomarkers for the diagnosis of tubal ectopic pregnancy.

## Introduction and background

An ectopic pregnancy (EP) is a pregnancy in which a fertilized ovum is implanted outside the uterine cavity with over 98% of the implantation in the fallopian tube. Tubal ectopic pregnancy remains the most common cause of maternal mortality and morbidity in the first trimester of pregnancy. EP affects approximately 1%–2% of pregnant women [1-2] and may seriously influence women's health and their future fertility, as tubal rupture is a common complication. Women with a previous history of the disease are at increased risk for future EP [3-6]. The pathogenesis of EP remains relatively unclear but it seems that an abnormal transport of the embryo through the tube, along with inflammation and an impaired tubal environment, may lead to ectopic implantation [7-11].

MicroRNAs (miRNAs) are small non-coding RNAs (containing about 21-24 nucleotides) found in plants, animals, and some viruses, and have emerged as regulators of overall gene expression representing another way of controlling stability and translation. Until now, more than a 1000 human miRNAs have been identified, each potentially controlling hundreds of targeted genes, while some of them are responsible for regulation of vital physiological events, including placental function and development. Several serum miRNAs were proposed as potential biomarkers of many diseases, including ectopic pregnancy [12-14].

The purpose of this review is to summarize the reports of the latest decade considering the use of miRNAs as biomarkers for diagnosis of ectopic pregnancy.

## Review

Ectopic pregnancy and the need for a reliable marker

Differentiation, migration, invasion, angiogenesis, proliferation, and apoptosis have a major participation in placental development which is critical for normal embryonic development. A variety of risk factors such as infection, surgery, smoking, and in vitro fertilization (IVF) have already been reported, while, on the other hand, the molecular biological processes (such as epigenetic mechanisms) are also believed to be involved in the pathogenesis of EP and include deoxyribonucleic acid (DNA) methylation, post-translational modification of histones, and small non-coding ribonucleic acids (RNAs) [15-23]. 

Unfortunately, there are currently no effective means for the prediction, prevention, or treatment of tubal implantation. Diagnosis is based on the combination of sonography and serum tests (serial human chorionic gonadotropin (hCG) and progesterone measurements) or surgical investigation in women with suspicious clinical symptoms (bleeding and/or pain). Despite the fact that serum hCG and progesterone have been the most intensively investigated biochemical markers, both seem to have low clinical utility in identifying women with EP, due to high false-positive and false-negative rates, and due to the lack of differentiation between hCG levels in EP and other fetal complications (stillbirth - spontaneous abortion). An increase in the hCG level of 53% in 48 hours is associated with viable intrauterine pregnancy, as suggested by the American College of Obstetricians and Gynecologists (ACOG) [24]. However, there is a crucial need for tests with high sensitivity—specifically for direct and reliable diagnoses of EP, since patients could experience life-threatening complications or unnecessary surgical and medical management (that may even result to the interruption of a potentially viable pregnancy) while waiting for the second blood sampling.

The role of miRNAs

MicroRNAs are small RNA fragments (18 to 25 nucleotides) that do not encode proteins, but act as post-transcriptional regulators of many genes by binding complementary sequences of RNAs, mainly located in the 3′-untranslated regions, whose expression or repression have a major participation on fetal development and pregnancy progress since the early stages of formation of the placenta. Each RNA sequence can target hundreds of genes, altering gene expression within a particular tissue or even causing an altered condition such as in many cancer types [6, 25-30]

To date, the interest on the role of miRNAs has dramatically expanded, as over 1,500 human miRNAs have been identified [31-34], and studies support that miRNAs are involved in the regulation of key biological phenomena, including cellular stemness and proliferation, apoptosis, prominently cancer, sepsis, autoimmune diseases and even cardiovascular and metabolic disorders in the obstetrics. MiRNAs (miR-21 and miR-141) expressed in placental tissue are also detected in high levels in maternal circulation, triggering the idea of being used as biomarkers for the prediction or even non-invasive diagnosis of preeclampsia (PE), intrauterine growth restriction and early pregnancy loss [35].

Studies on miRNAs and ectopic pregnancy

Since miRNAs are involved in embryo formation, development and implantation, and at the same time they are stable and detectable in maternal serum, their concentrations may reflect pathologic states of the tissue, with recent studies reporting adverse perinatal outcomes being accompanied by altered circulation levels of miRNAs.

Fu et al. reported in 2013 that miR-376c levels were downregulated in both placental and plasma samples at 16–18 weeks of pregnancy in 11 women who later developed PE, suggesting the possibility of using miR-376c as a predictive biomarker for PE [36].

Zhao et al. also tested the diagnostic value of miRNAs to EP by examining the relationship between serum pregnancy-associated markers, miRNAs, and early pregnancy outcomes. Of the 89 women who were presented to the emergency department (ED) with vaginal bleeding and/or abdominal pain/cramping that were diagnosed with viable intrauterine pregnancy (VIP), spontaneous abortion (SA), or EP, serum hCG and progesterone concentrations were determined by immunoassays. Serum miR-323-3p concentrations determined by real-time PCR were significantly elevated in EP and revealed more promising results than miR-517a, miR-519d, and miR-525-3p, with a 37% sensitivity rate (at a fixed-specificity of 90%) when used as a single marker. They also concluded that the combination of hCG and progesterone and miR-323-3p constitute a significant diagnostic accuracy with 96.3% sensitivity and 72.6% specificity [13].

In another study conducted in 2015 by Nagasaki University Graduate School of Biomedical Sciences in Japan, the researchers discovered that plasma concentration of cell-free pregnancy associated miRNAs—miR-323-3p, miR-515-3p, miR-517a, miR-517c, miR-518b—and the concentration of hCG have significant statistical differences in women with SA, EP and normal pregnancies (NP). Moreover, there was no significant statistical difference of cell-free serum concentration of miR-21 between three groups and they reported no relationship between serum levels of hCG and plasma cell free of miR-323-3p, miR-515-3p, miR-517a, miR-517c, miR-518b, and miR-21. Plasma cell-free concentration of miR-517a could distinguish ectopic pregnancy/ spontaneous abortion from natural pregnancy with a yield below the ROC curve of 0.9654 (95% coefficient interval 0.9172–1.0). In addition, plasma cell-free concentration of miR-323-3p could distinguish ectopic pregnancy from spontaneous abortion with a yield below the receiver operating characteristic (ROC) curve of 0.7454 ( 95% coefficient interval 0.5558 – 0.9349 ) [37].

Evidence about the great role of miRNAs in different clinical situations was found by the Lozoya et al. who studied the Lin28/Let-7 system in early human embryonic tissues and ectopic pregnancy. The Lin28 is an RNA binding protein which can bind to the precursors of Let7 miRNAs, blocking their capacity to interact with DICER, a miRNA enzyme that prevents maturation of miRNAs while Let7 participates in the regulation of LIN 28 expression, also controlled by other upstream elements, forming a regulatory hub that is involved in different processes. The aim of the research was to evaluate the expressions patterns of Lin28B messenger RNA (mRNA) and the related Let-7, mir-132, mir-145 in human embryonic tissue from early gestation between five to nine weeks of amenorrhea. Researchers also explored changes in the above targets in embryonic material from ectopic pregnancies at this early gestational window. In this study, the proposed biomarker mir-323-3p was also assayed in normal and ectopic pregnancies for comparative purposes. The results of this research were irritating with the authors reporting innovative information about miRNAs [38].

The expression of Lin28B for the early stage of gestation (≤6 weeks) was negligible, but maximum levels of Let7a, as well as mir-132, mir-145 were reported. The transitional week between weeks six to seven demonstrated a reciprocal change in the expression of the previous targets with a robust increase in Lin28B mRNA expression and a significant decrease of Let7a, and lesser extent of mir-132 and mir-145 levels. Lin28B expression levels demonstrate plateau between seven to nine weeks. On the other side, tubal ectopic implantation was correlated with quite reversal outcomes. The expression of Lin28B in early gestation (≤6weeks) was abnormally high and remained high in seven to nine weeks' embryonic tissue. Let7a miRNA expression was already suppressed in this time period (≤6 weeks) and remain suppressed from week seven and onwards as it normally occurred in normal pregnancies. Levels of mir-132, and mir-145 were not altered. This pattern of expression implicates an increased ratio of Lin28B/Let7a in embryonic tissue in ≤6 weeks ectopic gestations and it was hypothesized that this raise might favor a pro-proliferative and invasive phenotype and that the deregulation of Lin28B/Let7a system might contribute to the perturbation of thromboblast invasion commonly seen in ectopic pregnancies. Conclusively, the study reveals an increased ratio of Lin28B/Let7a in early pregnancy between six to seven weeks while high Lin28B/Let7a ratio was observed in ectopic embryonic tissue on ≤6 weeks of gestation. Another interesting factor concluded was the similar levels of expression of mir-323-3p in embryonic tissue of normal and ectopic pregnancies at ≤ 6 weeks. In seven to nine weeks of gestation, an increase was detected as it concerns the normal early pregnancies that was not observed in ectopic pregnancies at this age of gestation.

Until this research, this marker was identified only in plasma, and data of this effort cast doubts about the placental origin in ectopic pregnancies of the mir-32-3p levels, with authors supporting a non-embryonic origin of mir-32-3 while further investigation is proposed, in order to fully clarify the usefulness of this miRNA as marker in ectopic pregnancy [38].

From the aspect of implantation, a great role seems to be played by mir-212 and the regulation on the Olfactomedin 1 (OLFM1) and C-terminal-binding protein (CTBP); all of them are under the regulatory control of Luteinizing hormone (LH) / Follicle stimulation hormone (FSH) [39]. It is interesting that hCG induces the expression of mir-212 which in turn regulates the expression of OLFM1 and CTB1in mouse granulosa cells. Successful embryo implantation requires a synchronized dialogue between the blastocyst ant the receptive endometrium, that occurs in a limited time period known as “the implantation window” [40]. Recent studies show that downregulation of OLFM1in the endometrium and the fallopian tubes is associated with receptive endometrium and ectopic pregnancy [15, 41].

In 2015, Kottawwata et al. published a study in Biology of Reproduction journal, where the authors hypothesized that fetal hCG could increase the expression of mir-212 and downregulate the expression of OLFM1 and CTBP in favor of the embryo attachment to genital tract. The hCG suppressed proteins OLFM1 and CTBP1 in both human endometrial and fallopian epithelial cell lines used, however, hCG stimulated the expression of mir-212 and downregulated OLFM1 in both cell lines, but not CTBP1. Thus, hCG leads to downregulation of OLFM1 and CTBP1 expression in the fallopian and endometrium epithelial cell by stimulation of mir-212[42]. It is interesting that in the study of So et al., hCG through down-regulation of OLFM1 expression activated both extracellular-signal-regulated kinase (ERK) signaling pathway and the canonical Wnt (Wnt/β-catenin) pathway, resulting to the increase on spheroid attachment in vitro [43].

In another study, Dominguez et al. revealed a clear differential pattern of miRNA expression in embryonic tissues derived from normal and ectopic pregnancies by investigating a sample of 23 patients suffering from EP, and 29 normal pregnancies. Embryonic tissue samples were analyzed by real-time polymerase chain reaction (PCR) and miRNA microarray. Four miRNAs were differentially down-regulated (hsa-mir-196b, hsa-mir-30a, hsa-mir-873, and hsa-mir-337-3p) while three were upregulated (hsa-mir-1288, hsa-mir-451, and hsa-mir-223) in women suffering from EP compared to normal pregnancies. The authors proposed possible pathways of implementation and early placentation, but the real implication of these pathways in the ectopic pregnancy couldn’t be fully clarified [44].

Further evidence was provided in 2014 by Yi Feng et al. by examining the 23 patients suffering from EP. The authors showed that a significant decrease in DICER1 expression is accompanied by significant dysregulation of numerous miRNAs, including let-7i, miR-149, miR-182, and miR-424, in Fallopian tube tissues from the tubal implantation site of women with EP. Their findings not only provide a global miRNA expression profile but also identify four miRNAs that are specifically misexpressed in women with EP. They also show how irregular regulation of the specific and distinctive miRNA targets neural precursor cell expressed developmentally down-regulated protein 4 (NEDD4), TATA binding associated factor 15 (TAF15), and spen family transcriptional repressor (SPEN), might contribute to the onset of tubal pregnancy. Extracellular miRNAs are very stable and abundant in the circulation and they can be used as potential biomarkers for human diseases, including EP. Although several potential risk factors for EP have been proposed, further studies are needed to determine whether the alteration of miRNA biogenesis and subsequent changes in the regulation of miRNA targets are associated with women at risk for EP [45].

In 2017, Liu et al. presented a study where 21 miRNAs were analyzed in 36 patients with viable intrauterine pregnancy, 30 patients with spontaneous abortion (SA), and 34 patients with EP. Out of the 21 miRNAs, miR-873 and miR-223 were significantly lower in EP than in SA and VIP, while miR-323 was significantly higher in EP. For detecting EP, miR-873 presented the highest sensitivity as a single marker (61,76%) and combined with hCG and progesterone revealed a sensitivity of 79.41% (at a fixed specificity of 90%). Thus, the authors suggested miR-873 as a single, noninvasive and stable marker for early EP detection [46].

## Conclusions

Ectopic pregnancy affects approximately 1%–2% of pregnant women and may cause serious complications in maternal and fetal health and future fertility. The exact pathophysiology of EP is still unknown, however, it is firmly believed that retention of the embryo within the Fallopian tube due to impaired embryo-tubal transport combined to alterations in the tubal environment, lead to early implantation. Diagnosis of EP relies on a combination of transvaginal ultrasonographic, serum human chorionic gonadotropin and progesterone, however, due to the poor clinical utility for early detection, the development of a new, non-invasive serum test to diagnose EP with high sensitivity and specificity becomes mandatory in order to prevent both EP and the upcoming life-threatening complications, but also avoid unnecessary surgical or medical management that may interrupt a potentially viable pregnancy or affect patients’ fertility. A lot of investigations have focused their attention on this issue, and have reported many potential candidate biomarkers. The expression levels of several circulating miRNAs have shown to be novel biomarkers for the diagnosis of tubal EP with many advantages compared to the existing diagnostic tests. Serum miRNAs are relatively stable, with a clear differential pattern of expression in embryonic tissues of normal and ectopic pregnancies thus, it is firmly believed that miRNAs could reveal a novel diagnostic approach not only for diagnosis of EP but also for fetal monitoring during pregnancy. However, further studies are needed in order to reach safe conclusions.
